# Insomnia with objective short sleep duration is associated with longer duration of insomnia in the Freiburg Insomnia Cohort compared to insomnia with normal sleep duration, but not with hypertension

**DOI:** 10.1371/journal.pone.0180339

**Published:** 2017-07-26

**Authors:** Anna F. Johann, Elisabeth Hertenstein, Simon D. Kyle, Chiara Baglioni, Bernd Feige, Christoph Nissen, Alastair J. McGinness, Dieter Riemann, Kai Spiegelhalder

**Affiliations:** 1 Department of Psychiatry and Psychotherapy, Medical Center – University of Freiburg, Faculty of Medicine, University of Freiburg, Freiburg, Germany; 2 Medical Psychology and Medical Sociology, Faculty of Medicine, University of Freiburg, Freiburg, Germany; 3 Sleep and Circadian Neuroscience Institute (SCNi), Nuffield Department of Clinical Neuroscience, University of Oxford, Oxford, United Kingdom; University of Rome Tor Vergata, ITALY

## Abstract

**Study objectives:**

To replicate the association between insomnia with objective short sleep duration and hypertension, type 2 diabetes and duration of insomnia.

**Design:**

Retrospective case-control study.

**Setting:**

Department of Psychiatry and Psychotherapy, Medical Center—University of Freiburg.

**Participants:**

328 patients with primary insomnia classified according to DSM-IV criteria (125 males, 203 females, 44.3 ± 12.2 years).

**Interventions:**

N/A

**Measurements:**

All participants were investigated using polysomnography, blood pressure measurements, and fasting routine laboratory.

**Results:**

Insomnia patients with short sleep duration (< 6 hours) in the first night of laboratory sleep presented with a longer duration of insomnia compared to those with normal sleep duration (≥ 6 hours) in the first night of laboratory sleep. Insomnia patients who were categorised as short sleepers in either night were not more likely to suffer from hypertension (systolic blood pressure of ≥ 140 mm Hg, diastolic blood pressure of ≥ 90 mm Hg, or a previously established diagnosis). Data analysis showed that insomnia patients with objective short sleep duration were not more likely to suffer from type 2 diabetes (fasting plasma glucose level of ≥ 126 mg/dl, or a previously established diagnosis). However, the diabetes analysis was only based on a very small number of diabetes cases. As a new finding, insomnia patients who were categorised as short sleepers in either night presented with increases in liver enzyme levels.

**Conclusions:**

The finding on insomnia duration supports the concept of two distinct sub-groups of insomnia, namely insomnia with, and without, objectively determined short sleep duration. However, our data challenges previous findings that insomnia patients with short sleep duration are more likely to suffer from hypertension.

## Introduction

Insomnia is one of the most frequent mental disorders [1,2] leading to clinically relevant impairment in diverse areas of health-related quality of life [3] as well as to high societal cost [4]. Women have an increased risk to develop insomnia compared to men [5] and the prevalence of the disorder increases significantly with age [6]. Patients with insomnia have an increased risk of developing mental disorders, especially depression [7,8]. In the last decade, a number of studies suggest that insomnia also increases the risk for cardiometabolic diseases [9,10,11].

Recently, Vgontzas et al. [12] proposed two subtypes of insomnia with specific clinical and etiological characteristics. As such, insomnia with polysomnographically determined short sleep duration (total sleep time, TST < 6 hours) is characterised by a genetic predisposition, physiological hyperarousal, impaired neurocognitive function, longer duration of insomnia and an increased risk for cardiometabolic diseases. In contrast, insomnia with polysomnographically determined normal sleep duration (TST ≥ 6 hours) is characterised by a lack of physiological hyperarousal, sleep misperception, an anxious-ruminative profile, shorter duration of insomnia and no significant risk for cardiometabolic diseases.

With respect to the link to cardiometabolic diseases, Vgontzas et al. [13] investigated the association between insomnia with short sleep duration and hypertension using cross-sectional data from the Penn State Cohort comprising 1741 participants including 199 with insomnia. The results suggest that only individuals with both insomnia and short sleep duration are more likely to have hypertension in comparison with good sleeper controls. Based on the same cross-sectional dataset, Vgontzas et al. [14] reported that insomnia with short sleep duration is also associated with type 2 diabetes. Using longitudinal data from the same cohort, insomnia patients with short sleep duration were also characterised by a longer duration of insomnia [15], an increased risk for hypertension [16], and an increased all-cause mortality [17]. Of note, these analyses were based on one night of polysomnography (PSG) only.

Apart from the seminal findings based on the Penn State Cohort, only limited evidence directly supports that insomnia with short sleep duration is specifically related to cardiometabolic diseases and longer duration of insomnia; only one study directly replicated the hypertension finding [18]. In light of the important clinical implications, the current study aimed at replicating three central findings of the Penn State Cohort, namely the association between insomnia with short sleep duration and hypertension, type 2 diabetes and duration of insomnia in a clinical sample of patients with insomnia.

Furthermore, based on the hypothesis that insomnia with short sleep duration manifests primarily in the biological domain [12], the current study extends previous research by investigating basic laboratory parameters in the proposed sub-groups of insomnia. Thus, another aim of this study was to explore the potential association between insomnia with short sleep duration and several laboratory parameters (erythrocytes, haemoglobin, haematocrit, erythrocyte mean corpuscular volume (MCV), leukocytes, thrombocytes, creatinine, alanine aminotransferase (ALT), gamma glutamyl transferase (γGT), thyroid stimulating hormone (TSH)) that are routinely assessed in our sleep disorders centre.

## Material and methods

### Participants

The study sample was obtained by reviewing data of an archival database of the sleep laboratory of the Department of Psychiatry and Psychotherapy, University Medical Centre Freiburg (Freiburg Insomnia Cohort). In total 328 clinically referred patients with primary insomnia according to DSM-IV-TR criteria [19] were included in the present analysis (203 women; 125 men; mean age 44.3 ± 12.2 years). All patients were investigated between 1995 and 2014. Overall, in this period, 4897 individuals were investigated in the sleep laboratory. In accordance with DSM-5 [20], all patients with a sleep-related complaint of non-restorative sleep only or with an insomnia duration of less than 3 months were excluded from the current study. The frequency criterion of DSM-5, however, could not be confidently determined in all patients from the medical records.

A clinical interview by an experienced psychiatrist and PSG were used to identify and exclude patients with psychiatric disorders or occult sleep disorder pathology (including hypersomnia, parasomnia, sleep-related breathing disorder, sleep-related movement disorder and circadian rhythm sleep disorder). In addition, routine fasting laboratory investigations (blood cell count, liver, renal and thyroid function) in the morning were used to exclude those with serious medical conditions. All participants were free of any psychoactive medication at least 1 week prior to the sleep laboratory examination. Participants with antihypertensive, diabetes or thyroid medication were not excluded from the current study. During the two nights of examination in our sleep laboratory, patients had to refrain from alcohol and caffeine. All patients were asked to complete the Pittsburgh Sleep Quality Index (PSQI) [21], a 19-item measure of different aspects of sleep quality, and the Beck depression inventory [22], a 21-item measure of depression levels.

The study was conducted in accordance with the Declaration of Helsinki. The study protocol was approved by the Institutional Review Board of the University Medical Centre Freiburg. Written consent was taken from all patients prior to the examination in the sleep laboratory, allowing us to analyse their data in retrospective studies.

### Polysomnography

All patients underwent two consecutive nights of PSG sleep monitoring. Sleep was recorded for eight hours from 22:24 h ± 27 min until 6:24 h ± 27 min adjusted to individual habitual bedtimes. All recordings included EEG (C3-A2; C4-A1), EOG (horizontal and vertical) and EMG (submental), and were scored visually by experienced raters according to the AASM criteria [23]. All patients were screened for apneas and periodic leg movements by monitoring abdominal and thoracic effort, nasal airflow, oxymetry, and bilateral tibialis anterior EMG. Sleep recordings were evaluated for the following parameters of sleep continuity: total sleep time (TST); sleep efficiency (ratio of TST to time in bed); sleep onset latency defined as time from lights out until sleep onset (defined as first epoch of stage 2); wake after sleep onset (WASO) defined as difference between sleep period time (SPT; time from sleep onset until final awakening) and TST; number of awakenings; and arousal index (the number of arousals per hour). Sleep architecture parameters were percentages of stages 1, 2, slow wave sleep (SWS) and rapid eye movement sleep (REM) referred to SPT.

### Key measurements

Polysomnographic TST was used to divide patients into two groups, namely insomnia patients with short sleep duration (TST < 6 hours) and insomnia patients with normal sleep duration (TST ≥ 6 hours). Classification used to allocate patients to short (< 6) or the normal sleep (≥ 6 hours) sub-group was based on two consecutive nights of PSG sleep monitoring. Consequently, patients assigned to the sub-group short sleep (based on the first night) may differ from patients assigned to the sub-group short sleep (based on the second night). Blood pressure was routinely measured by trained nurses in the morning after awakening, and a resting period. Until April 2010, this was done manually, since April 2010 digitally (Boso Medicus Uno; Boso Germany; http://www.boso.de/). Hypertension was defined as a systolic blood pressure of ≥ 140 mm Hg, a diastolic blood pressure of ≥ 90 mm Hg [24], antihypertensive medication, or a previously established diagnosis. Routine fasting laboratory samples were taken from all patients in the morning after the first sleep laboratory night. Type 2 diabetes was defined as a fasting plasma glucose level of ≥ 126 mg/dl [25], diabetes medication, or a previously established diagnosis. For exploratory purposes, laboratory samples were also analysed with respect to erythrocytes, haemoglobin, haematocrit, mean corpuscular volume (MCV), leukocytes, thrombocytes, creatinine, alanine aminotransferase (ALT), γ-glutamyl transpeptidase (γGT), and thyroid-stimulating hormone (TSH).

### Statistical analysis

Group differences in demographic and polysomnographic data were investigated using two-sample t-tests and chi-squared tests. The impact of the insomnia sub-group (short vs normal sleep duration) on hypertension, type 2 diabetes and insomnia duration was investigated using logistic regression analyses (hypertension, type 2 diabetes) and linear regression analyses (insomnia duration). For these analyses, three different models were applied. Model 1 included age and sex as covariates. Model 2 included the covariates of model 1 as well as the sleep apnea index from the first night as additional covariate. Model 3 included the covariates of model 2 as well as BMI and BDI scores as additional covariates. Exploratory analyses of laboratory parameters were conducted using one-way ANOVAs with age and sex as covariates. For these analyses, liver enzymes were logarithmically transformed because of highly skewed distributions. In addition, the analysis of TSH was restricted to individuals without thyroid medication. Of note, all analyses were carried out separately for both classifications (short vs. normal sleep subgroup) for both nights (first and second night). The level of significance was set at p < 0.05 (two-tailed) for all analyses. All analyses were carried out using the statistical software package R (http://www.R-project.org/).

## Results

### Sample characteristics

The total sample of 328 patients had a mean age of 44.3 ± 12.2 years, a mean BMI of 23.8 ± 3.6 kg/m^2^, a mean score of 11.2 ± 3.2 on the PSQI, and a mean score of 6.4 ± 4.5 on the BDI (after exclusion of the two sleep-related items). Demographic characteristics of the study sample, when classifying the group into insomnia patients with short sleep duration and insomnia patients with normal sleep duration, are presented in Table 1. Of note, 47 patients had a TST < 6 hours in both nights. Insomnia patients with short sleep duration were significantly older than insomnia patients with normal sleep duration (classification based on the first night: t = 5.35; p < 0.001; classification based on the second night: t = 5.08; p < 0.001). No other significant group differences were apparent for either classification. According to the clinical interview, seventeen patients suffered from sleep-onset insomnia, 116 from sleep-maintenance insomnia, and 189 from mixed insomnia. In 6 patients, the type of insomnia could not be determined retrospectively. Nine patients suffered from type 2 diabetes. Out of these, 3 patients were on diabetes medication (two of them on metformin, and one on metformin and sitagliptin) and 8 patients had a fasting plasma glucose level of > 125mg/dl. Thus, 2 patients presented with a fasting plasma glucose level of > 125mg/dl despite being on diabetes medication. Ninety-seven patients suffered from hypertension. Out of these, 25 patients received antihypertensive medication and 83 patients had either a diastolic blood pressure > 90 mm Hg or a systolic blood pressure > 140 mm Hg (33 patients with > 90 mm Hg and 73 patients with > 140 mm Hg). Thus, 11 patients presented either with a diastolic blood pressure > 90 mm Hg or a systolic blood pressure > 140 mm Hg despite being on antihypertensive medication.

**Table 1:** Description of the study population (means ± standard deviations).

	Short sleep (1. night)	Normal sleep (1. night)	t/χ^2^	Short sleep (2. night)	Normal sleep (2. night)	t/χ^2^
Sex (M/F)	61/87	64/116	0.88	23/41	102/162	0.07
Age (years)	48.1 ± 11.7	41.2 ± 11.8	5.35***	51.4 ± 12.6	42.6 ± 11.5	5.08***
BMI (kg/m^2^)	23.9 ± 3.5	23.7 ± 3.7	0.43	24.0 ± 4.1	23.8 ± 3.4	0.37
PSQI	11.5 ± 3.1	10.9 ± 3.3	1.68	11.7 ± 2.9	11.1 ± 3.3	1.56
BDI	6.1 ± 4.1	6.6 ± 4.9	-1.07	6.1 ± 4.6	6.4 ± 4.5	-0.50

BMI: body mass index; PSQI: Pittsburgh Sleep Quality Index; BDI: Beck Depression Inventory;

***: p < 0.001.

### Polysomnography

Polysomnographic data are presented in Table 2 (first night) and Table 3 (second night). By design, there were pronounced differences between insomnia patients with short sleep duration and insomnia patients with normal sleep duration in terms of sleep continuity and sleep architecture of both nights. Of note, when the second night was used for sub-group classification, the sleep apnea index in the first night was greater in those with short sleep duration compared to those with normal sleep duration.

**Table 2:** Polysomnographic data from the first sleep laboratory night (means ± standard deviations).

	Short sleep (1. night)	Normal sleep (1. night)	t	Short sleep (2. night)	Normal sleep (2. night)	t
TST (min)	302.8 ± 41.9	407.0 ± 28.3	-25.80***	318.0 ± 64.7	370.2 ± 57.8	-5.90***
Sleep efficiency (%)	63.2 ± 8.8	84.7 ± 5.9	-25.61***	66.3 ± 13.4	77.1 ± 12.0	-5.92***
SOL (min)	35.5 ± 26.6	18.7 ± 12.8	7.01***	30.0 ± 22.0	25.4 ± 21.8	1.50
WASO (min)	117.2 ± 47.3	48.8 ± 24.6	15.90***	108.4 ± 55.5	72.7 ± 46.0	4.77***
NOA	35.4 ± 17.7	29.4 ± 12.5	3.46***	36.9 ± 16.8	31.0 ± 14.8	2.58*
Arousal index (h^-1^)	21.7 ± 8.4	17.6 ± 7.7	4.50***	21.5 ± 8.3	19.0 ± 8.2	2.17*
Sleep apnea index (h^-1^)	0.5 ± 0.9	0.5 ± 1.0	0.11	1.0 ± 1.5	0.4 ± 0.7	2.97**
PLMS arousal index (h^-1^)	0.6 ± 1.5	0.6 ± 1.4	0.02	0.5 ± 1.1	0.6 ± 1.5	-0.51
Stage 1 (% SPT)	11.0 ± 5.3	9.8 ± 5.1	2.07*	10.9 ± 5.0	10.2 ± 5.3	0.95
Stage 2 (% SPT)	44.8 ± 9.6	55.4 ± 6.6	-11.37***	45.0 ± 11.6	51.9 ± 8.6	-4.46***
SWS (% SPT)	3.6 ± 5.1	5.4 ± 6.3	-2.87**	4.2 ± 5.7	4.7 ± 5.9	-0.64
REM (% SPT)	13.1 ± 5.0	18.6 ± 4.6	-10.29***	14.5 ± 5.4	16.5 ± 5.5	-2.57*

TST: total sleep time; SOL: sleep-onset latency; WASO: wake after sleep onset; NOA: number of awakenings; PLMS: periodic leg movements during sleep; SWS: slow wave sleep; REM: rapid eye movement sleep;

*: p < 0.05;

**: p < 0.01;

***: p < 0.001.

**Table 3:** Polysomnographic data from the second sleep laboratory night (means ± standard deviations).

	Short sleep (1. night)	Normal sleep (1. night)	t	Short sleep (2. night)	Normal sleep (2. night)	t
TST (min)	368.3 ± 56.8	411.3 ± 37.3	-7.90***	307.8 ± 45.8	412.3 ± 26.1	-17.57***
Sleep efficiency (%)	76.8 ± 11.9	85.6 ± 7.8	-7.72***	64.2 ± 9.6	85.9 ± 5.4	-17.44***
SOL (min)	20.3 ± 19.4	15.7 ± 11.1	2.58*	25.1 ± 24.7	16.0 ± 11.8	2.89**
WASO (min)	77.8 ± 51.8	46.6 ± 32.7	6.35***	126.5 ± 51.8	44.8 ± 24.0	12.31***
NOA	34.4 ± 17.3	27.6 ± 12.6	4.05***	38.5 ± 20.3	28.8 ± 13.1	3.66***
Arousal index (h^-1^)	17.7 ± 6.6	15.5 ± 6.5	2.93**	18.7 ± 6.9	16.0 ± 6.5	2.91**
Stage 1 (% SPT)	9.3 ± 4.3	8.6 ± 4.5	1.42	9.7 ± 4.1	8.8 ± 4.5	1.62
Stage 2 (% SPT)	50.4 ± 10.7	54.9 ± 7.6	-4.24***	42.0 ± 9.6	55.5 ± 7.1	-10.57***
SWS (% SPT)	4.8 ± 6.4	6.0 ± 6.6	-1.61	4.6 ± 6.3	5.6 ± 6.6	-1.16
REM (% SPT)	17.8 ± 4.9	20.2 ± 4.6	-4.49***	15.0 ± 4.3	20.1 ± 4.5	-8.47***

TST: total sleep time; SOL: sleep-onset latency; WASO: wake after sleep onset; NOA: number of awakenings; SWS: slow wave sleep; REM: rapid eye movement sleep;

*: p < 0.05;

**: p < 0.01;

***: p < 0.001.

### Hypertension

In the full sample, 97 patients (31.5%) had hypertension (35.0% of those with short sleep duration in the first night; 28.6% of those with normal sleep duration in the first night; 43.5% of those with short sleep duration in the second night; and 28.5% of those with normal sleep duration in the second night).

Model 1 (including age, and sex as covariates) did not show any significant association between short sleep duration and hypertension in comparison with normal sleep duration (sub-group classification based on the first night: OR 0.79, CI 0.46–1.36; sub-group classification based on the second night: OR 1.21, CI 0.64–2.29).

Likewise, Models 2 (including the covariates of model 1 as well as the sleep apnea index from the first night as additional covariate) and 3 (including the covariates of model 2 as well as BMI and BDI scores as additional covariates), did also not show any significant association between short sleep duration and hypertension (Model 2, sub-group classification based on the first night: OR 0.90, CI 0.51–1.59; sub-group classification based on the second night: OR 1.46, CI 0.74–2.88; Model 3, sub-group classification based on the first night: OR 0.80, CI 0.41–1.55; sub-group classification based on the second night: OR 1.82, CI 0.82–4.02).

### Type 2 diabetes

In the full sample, 9 patients (3.8%) had type 2 diabetes (4.7% of those with short sleep duration in the first night; 3.1% of those with normal sleep duration in the first night; 7.7% of those with short sleep duration in the second night; and 2.7% of those with normal sleep duration in the second night).

Model 1 (including age, and sex as covariates) did not show any significant association between short sleep duration and type 2 diabetes in comparison with normal sleep duration (sub-group classification based on the first night: OR 1.40, CI: 0.35–5.58; sub-group classification based on the second night: OR 2.93, CI: 0.70–12.26).

Likewise, Models 2 (including the covariates of model 1 as well as the sleep apnea index from the first night as additional covariate) and 3 (including the covariates of model 2 as well as BMI and BDI scores as additional covariates), did also not show any significant association between short sleep duration and type 2 diabetes (Model 2, sub-group classification based on the first night: OR 1.54, CI: 0.39–6.15; sub-group classification based on the second night: OR 2.57, CI 0.57–11.49; Model 3, sub-group classification based on the first night: OR 1.39, CI 0.34–5.67; sub-group classification based on the second night: OR 2.30, CI 0.48–10.96).

### Insomnia duration

Insomnia duration was 11.3 ± 11.0 years in the entire sample (13.3 ± 11.9 years in those with short sleep duration in the first night; 9.6 ± 9.8 years in those with normal sleep duration in the first night; 13.0 ± 13.0 years in those with short sleep duration in the second night; and 10.9 ± 10.4 years in those with normal sleep duration in the second night).

Model 1 (including age, and sex as covariates) showed a significantly increased insomnia duration in those with short sleep in the first night in comparison to those with normal sleep in the first night (β = 1.99; F = 9.03; p = 0.003), but no significant group difference when sub-groups were classified according to the second night (β = -0.47; F = 1.90; p = 0.169).

The significant group difference persisted in model 2 and model 3 when sub-groups were classified according to the first night (model 2: β = 1.68; F = 6.39; p = 0.012; model 3: β = 2.22; F = 6.02; p = 0.015), as did the lack of significant group difference for models 2 and 3 when sub-groups were classified according to the second night (model 2: β = -1.15; F = 1.66; p = 0.199; model 3: β = 0.18; F = 3.34; p = 0.069).

**Fig 1:**
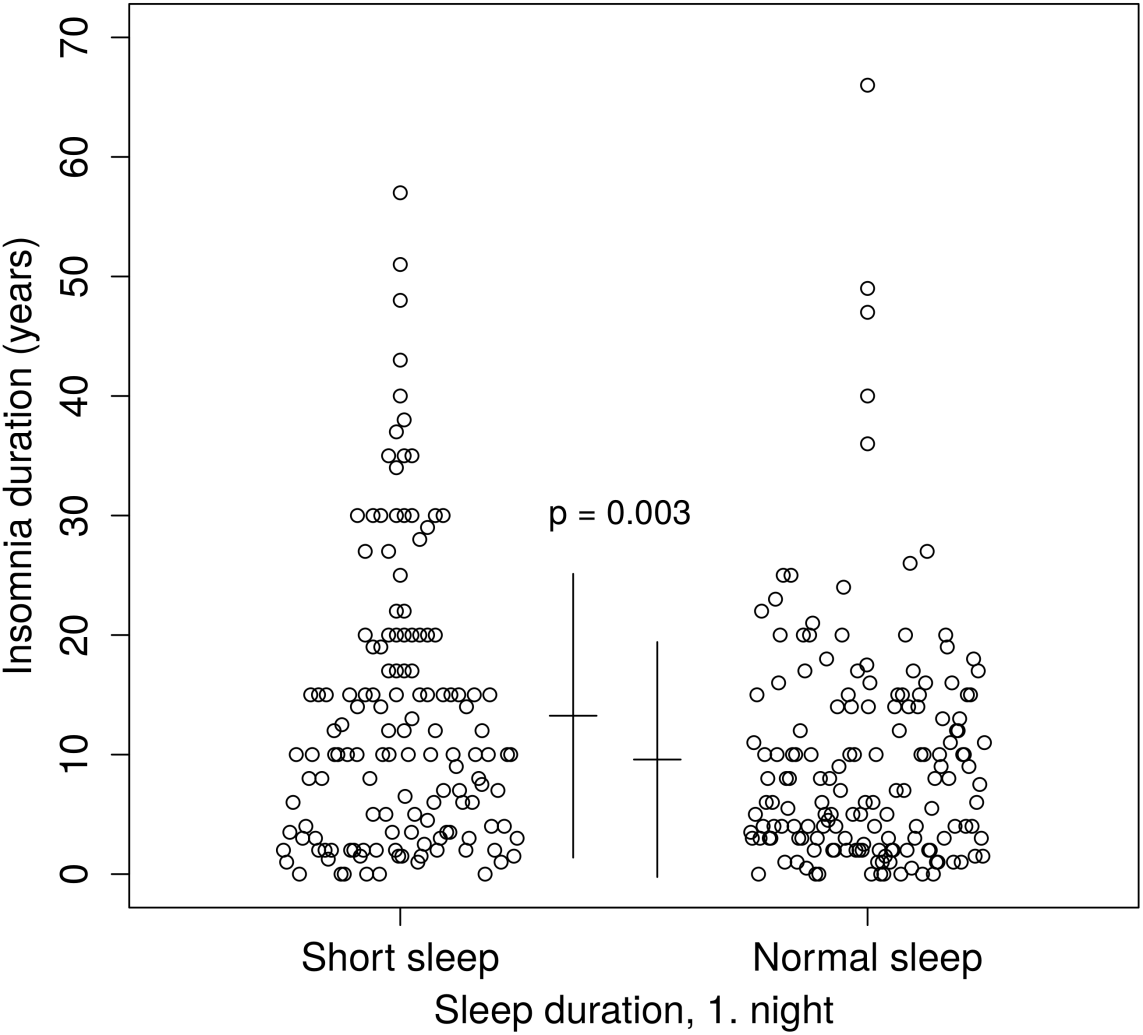
Insomnia duration for insomnia patients with short and normal sleep duration in the first sleep laboratory night. Insomnia patients with short sleep duration had a significantly longer insomnia duration than insomnia patients with normal sleep duration (p = 0.003 for model 1).

Insomnia duration for those with short sleep duration and normal sleep duration according to the first sleep laboratory night are presented in Fig 1.

### Routine laboratory parameters

The results of the routine laboratory parameters are presented in Table 4. Levels of both liver enzymes (ALT, γGT) were higher in insomnia patients with short sleep duration compared to those with normal sleep duration, both when sub-groups were classified according to the first and second night. These group differences were relatively stable when controlling for alcohol use (sub-group classification based on the first night: ALT: β = 0.10; F = 6.31, p = 0.013; γGT: β = 0.11; F = 6.68, p = 0.010; sub-group classification based on the second night: ALT: β = 0.16; F = 4.62, p = 0.033; γGT: β = 0.13; F = 3.50, p = 0.063).

**Table 4:** Routine laboratory parameters (means ± standard deviations).

	n	Short sleep (1. night)	Normal sleep (1. night)	F	Short sleep (2. night)	Normal sleep (2. night)	F
Erythrocytes (x 10^6^/μl)	292	4.81 ± 0.43	4.73 ± 0.41	5.15*	4.78 ± 0.37	4.76 ± 0.43	0.15
Haemoglobin (g/dl)	292	14.4 ± 1.3	14.1 ± 1.3	6.27*	14.3 ± 1.4	14.2 ± 1.3	0.57
Haematocrit (%)	292	42.6 ± 3.5	41.9 ± 3.3	5.72*	42.6 ± 3.4	42.1 ± 3.4	2.05
MCV (fl)	292	88.8 ± 5.3	88.7 ± 3.6	0.02	89.5 ± 6.5	88.5 ± 3.7	2.21
Leukocytes (x 10^3^/μl)	292	5.92 ± 1.39	5.94 ± 1.56	0.01	5.96 ± 1.39	5.92 ± 1.51	0.03
Thrombocytes (x 10^3^/μl)	291	246 ± 57	248 ± 55	0.06	249 ± 57	247 ± 56	0.11
Creatinine (mg/dl)	237	0.84 ± 0.14	0.84 ± 0.15	0.14	0.87 ± 0.16	0.83 ± 0.14	3.53
ALT (U/l)	247	23.1 ± 16.8	19.2 ± 11.7	5.85*	23.9 ± 13.0	20.1 ± 14.5	6.67*
γGT (U/l)	250	25.3 ± 26.8	17.8 ± 10.2	6.65*	23.7 ± 15.3	20.4 ± 20.6	7.01**
TSH (μU/ml)	155	2.01 ± 1.31	1.80 ± 0.87	1.45	1.93 ± 1.41	1.88 ± 0.97	0.05

MCV: erythrocyte mean corpuscular volume; ALT: alanine aminotransferase; γGT: gamma glutamyl transferase; TSH: thyroid stimulating hormone;

*: p < 0.05;

**: p < 0.01.

In addition, erythrocytes, haemoglobin and haematocrit were significantly increased in insomnia patients with short sleep duration in the first night in comparison to those with normal sleep duration in the first night. This difference was not evident when sub-groups were classified according to the second night.

## Discussion

The results of the current Freiburg Cohort corroborate previous findings [15] that insomnia with objective short sleep duration is characterised by a longer duration of insomnia in comparison with insomnia with normal sleep duration. In detail, those with short sleep duration in the first sleep laboratory night had a 3.7 years longer insomnia duration in comparison with those with normal sleep duration. Moreover, as a new finding, insomnia patients with objective short sleep duration appear to present with increases in liver enzyme levels. However, we did not find any support for the assumption that insomnia with objective short sleep duration is specifically associated with hypertension, a finding that has been reported in studies based on the Penn State Cohort [13,14].

Several differences between our Freiburg Cohort and the Penn State Cohort may explain the discordance of the results. First, the current sample included fewer insomnia patients with hypertension and type 2 diabetes than the Penn State Cohort, in which these disorders were purposefully oversampled. This results in a reduction of statistical power in the current study, which is particularly evident for the analysis of type 2 diabetes. In the course of the analysis it turned out that only 9 patients with insomnia presented with type 2 diabetes in the current sample compared to 36 patients with insomnia in the Penn State Cohort. Because of this and other limitations of the current analyses of type 2 diabetes (see below in the limitations section), the non-significant results on the association between sleep duration and type 2 diabetes in those with insomnia should be regarded as non-conclusive. In the analysis of hypertension, we included 97 and the Penn State Cohort 103 patients with insomnia and hypertension. With respect to statistical power, it is also important to note that we included more insomnia patients than the Penn State Cohort (328 vs. 199), while the Penn State Cohort had a substantially larger overall sample size with 1741 community-sampled individuals including healthy subjects and individuals with poor sleep who did not meet diagnostic criteria for insomnia. Possibly, subjective insomnia severity, as well as polysomnographically determined SOL and WASO were also different between the cohorts, however, details were not reported in Vgontzas et al. [13] and Vgontzas et al. [14].

Second, in our study, patients underwent two nights of PSG sleep monitoring, whereas the Penn State Cohort included only one night. It is, however, unlikely that this difference accounts for the discrepancy in the results on hypertension and type 2 diabetes as we have provided analyses for the first night that are equivalent to those of Vgontzas et al. [13,14]. With respect to duration of insomnia, our results replicate the ones by Vgontzas et al. [15], i.e. we found a negative association between TST in the first sleep laboratory night and insomnia duration. Insomnia patients with objective short sleep duration may, thus, be particularly prone to a longer duration of insomnia because they suffer from a more severe form of the disorder. However, we did not find this association for the second sleep laboratory night. Thus, this pattern of results may be best explained by the assumption that sleep reactivity [26], i.e. the tendency to show sleep disturbance when facing a stressful sleep challenge like the first night in a sleep laboratory [27], is a maintaining factor of insomnia, and, thus, leads to a longer duration of insomnia.

Third, it is not entirely clear whether the publications of the Penn State Cohort are based on exploratory or confirmatory data analyses with a priori hypotheses. This is of particular importance as exploratory analyses are associated with an increased risk of false-positive findings, especially when a large number of significance tests were conducted [28]. In particular, varying criteria for sub-grouping is rather typical for exploratory than for confirmatory data analysis (compare [13,16,29]).

In general, it should be noted that our clinical sample appears to be representative of the population of insomnia patients who are investigated in specialised sleep medicine centres. This is suggested by the mean PSQI score (11.2), which is in the range of previous studies on insomnia [30,31,32]. Moreover, comparing the PSG data of the current sample with a recent meta-analysis on polysomnographic sleep in insomnia [33] suggests that our sample is representative in terms of objective TST (391 vs. 392 min) and SOL (20 vs. 18 min).

The exploratory finding of increased liver enzymes in those with insomnia and short sleep duration is a new finding. Of particular importance, this finding holds true even when controlling for alcohol consumption. As alcohol consumption has been assessed subjectively without objective validation, and we could not control for lifetime history of hypnotic use, it is, however, still conceivable that this finding relates to an increased substance use in those patients with objective short sleep duration. Moreover, it should be noted that most ALT and γGT values were in the normal range. Nevertheless, this finding may stimulate further research into the association of objective sleep duration and liver diseases in individuals with insomnia. In addition, we found increases in red blood cells and haemoglobin/haematocrit in insomnia patients with short sleep duration in the first night. However, as this finding was not evident when sub-groups were classified according to the second night, this finding should be replicated before firm conclusions can be drawn.

Four limitations of the current study need to be addressed. First, we have analysed data from routine clinical practice which might have been assessed in a less standardised manner than the one of prospective clinical studies. For example, blood pressure was measured manually in some individuals and digitally in others. This may have increased the error variance in the corresponding analyses. Second, the Freiburg Insomnia Cohort was healthier than the Penn State Cohort including fewer insomnia patients with hypertension and type 2 diabetes. This has probably reduced the statistical power for several analyses. However, while both samples may be considered as small, it should also be noted that we had a larger sample of insomnia patients than the Penn State Cohort. Third, we did not have any information about the history of hypoglycemic events in our study participants or data on long-term blood glucose levels. Finally, as the study had a cross-sectional design, conclusions about causality cannot be drawn.

In summary, both the Penn State Cohort and the Freiburg Insomnia Cohort reported a significant negative association between total sleep time in the first sleep laboratory night and insomnia duration. This supports the assumption that insomnia can be divided into distinct sub-groups as suggested by Vgontzas et al. [12]. However, our new data challenge the findings of the Penn State Cohort that insomnia patients with short sleep duration are more likely to suffer from hypertension. Thus, these findings of the Penn State Cohort may still be considered preliminary observations which should be further investigated. Our exploratory finding, the association between insomnia with objective short sleep duration and increased liver enzymes awaits further testing and validation.
